# Supplementary material for: Childcare attendance and risk of infectious mononucleosis: A population-based Danish cohort study
Source: PLoS One. 2021 Dec 22;16(12):e0261665. doi: 10.1371/journal.pone.0261665 (PMC8694440; doi:10.1371/journal.pone.0261665)
Supplement: Attachment — Submitted filename: Response to Reviewers..docx [file pone.0261665.s001.docx]

Answers to reviewer comments

Reviewer #1: Very nice data and approach to the problem of looking at childcare attendance and IM risk.

As a non-statistician and.or epidemiologist I don't feel qualified to comment on how you prepared your data for analysis. The number of assumptions about what to include and what to exclude is concerning, but you have made attempts to justify why you have done this. However, the data is what it is and there are not many countries that can do this sort of study. Well done on getting this far. Reading your paper has made me think quite deeply about EBV and IM risk.

ANSWER: Thank you very much for these comments.

Reviewer #2: Dear Editor
The manuscript is interesting, trying to answer important clinical points for the first time. In addition, I think it is well written; therefore I suggest it should be accepted for publication.

ANSWER: Thank you very much for these comments.

Reviewer #3: Overall the paper is well written. However, the abstract should nevertheless be revised with respect to language.

ANSWER: Thank you for pointing out this weakness. The abstract has been revised in 7 places to improve the grammar. Furthermore the sentence ”Hazard ratios…were obtained …. adjusted for sibship structure, parental ethnicity and maternal age” was revised to ”Hazard ratios…were obtained …. adjusted for sibship structure, and sociodemographic variables including parental ethnicity and maternal age” to be thoroughly correct.

The statistical analysis is appropriate but the presentation is difficult to follow. A reason for this is that everything is presented in form of tables. Instead, HRs can be visualized see e.g. a turorial (for R)

This will improve the presentation and allows the reader to obtain a better overview. I would present the visualization in addition to the tables.

ANSWER: We have added a figure presenting the rightmost column of the table as a forest plot – with abbreviated headings. As the last sentence of ”Statistical methods” we have added: ”Fig 1 was prepared using the forestplot package in R, to visually augment Table 1.” The figure is referenced once in the second paragraph of the results section: ”The effect of sibship structure (sibling age differentials and having 0-3-year-old sibs) was broadly as expected; i.e., more protection the smaller the difference in age, younger sibs being generally more protective than older sibs and a marked instantaneous effect of having 0-3-year-old sibs on IM risk (see [1]) (Table 1, Fig 1).”

Furthermore we have completed the formatting for PLoS, corrected a few grammatical errors and made some slight additions to a sentence in the introduction: ”Presumably EBV is transmitted through saliva (including sneezing [22]) to the pre-adolescents infected, because most other suggested routes of transmission (transfusion, sex, deep kissing) would seem less relevant for children, while transmission routes of particular relevance to children (breast milk, in utero infection) appear to play a minor role [7,8,23–26]”, and a sentence in the beginning of the Discussion section: ”However, the effect sizes we found regarding a phenomenon mainly caused by EBV infection are so small as to have no public health implications”.
